# Changing Patterns of Hospitalisation and Orthopaedic Procedure Profiles in Major Inflammatory Rheumatic Diseases in Germany: a Nationwide Analysis of Hospital Discharge Data

**DOI:** 10.1007/s00296-026-06247-5

**Published:** 2026-07-11

**Authors:** Houmam Anees, Reem Jamous, Christian Heiss, Thaqif El Khassawna, Christoph Biehl

**Affiliations:** 1 Department of Trauma, Hand and Reconstructive Surgery, Faculty of Medicine, Justus-Liebig-University of Giessen, 35392 Giessen, Germany; 2 Experimental Trauma Surgery, Faculty of Medicine, Justus-Liebig-University of Giessen, 35392 Giessen, Germany; 3 Biruni University, Istanbul, Turkey; 4 School of Pharmacy, The University of Jordan, Amman, 11942 Jordan

**Keywords:** Rheumatoid arthritis, Arthritis, Psoriatic arthritis, Spondylitis, Ankylosing spondylitis, Juvenile idiopathic arthritis, Health services utilisation, Arthroplasty, Epidemiology

## Abstract

Inflammatory rheumatic diseases are major contributors to morbidity and healthcare utilisation worldwide. Advances in disease-modifying antirheumatic drug therapy have substantially changed disease management and may have influenced patterns of inpatient care and orthopaedic surgical treatment. However, nationwide data on long-term hospitalisation trends remain limited. This nationwide analysis of aggregated inpatient hospital discharge data was based on data from the German Federal Health Monitoring system (GBE-Bund) between 2010 and 2023. Hospitalisations associated with rheumatoid arthritis (RA), psoriatic arthritis (PsA), ankylosing spondylitis (AS), juvenile idiopathic arthritis (JIA), and osteomyelitis were identified using ICD-10-GM codes. Orthopaedic procedures were analysed using diagnosis-related group data from the German Institute for the Hospital Remuneration System (InEK). Hospitalisation trends differed substantially across disease groups. RA hospitalisations declined from 29,775 cases in 2010 to 24,992 cases in 2023 (− 16.1%), while JIA showed the largest reduction (− 43.0%). In contrast, PsA hospitalisations increased (+ 7.6%), whereas AS remained largely stable (+ 2.9%). Osteomyelitis, included as a comparator musculoskeletal disorder, showed an overall stable pattern. A marked reduction in hospitalisations was observed across all disease groups in 2020. Age-standardised hospitalisation rates largely mirrored these findings, with RA decreasing from 40 to 30 and JIA from 7 to 4 hospitalisations per 100,000 population between 2010 and 2023. Distinct orthopaedic procedure profiles were identified, with knee and hip replacement procedures predominating in RA and spine-related interventions predominating in AS. Nationwide hospitalisation patterns in inflammatory rheumatic diseases have changed substantially over the past decade. Despite advances in medical therapy, orthopaedic surgical interventions continue to represent an important component of disease management, highlighting the continued need for integrated rheumatologic and orthopaedic care.

**Supplementary Information:**

The online version contains supplementary material available at 10.1007/s00296-026-06247-5.

## Introduction

Inflammatory rheumatic diseases represent a major cause of morbidity and healthcare utilisation worldwide. Among the most prevalent conditions are rheumatoid arthritis, psoriatic arthritis, and ankylosing spondylitis, which are characterised by chronic inflammation leading to progressive joint and structural damage. If insufficiently controlled, these diseases can result in significant functional impairment, reduced quality of life, and increased need for surgical intervention [1].

Over the past two decades, the management of inflammatory rheumatic diseases has undergone substantial transformation. The introduction of biologic and targeted synthetic disease-modifying antirheumatic drugs (DMARDs) has markedly improved disease control and reduced inflammatory activity in many patients. These therapeutic advances have been associated with improvements in disease control and reduced radiographic progression. As a consequence, these changes may have influenced the need for hospital-based care and orthopaedic surgical interventions, although findings across studies have been heterogeneous [2].

Previous studies have suggested a decline in joint replacement surgery among patients with rheumatoid arthritis in the era of modern pharmacological treatment [3]. Although results vary across populations, many studies are limited to specific regions or cohorts, and direct comparisons across different inflammatory rheumatic diseases remain scarce. Furthermore, less is known about nationwide trends in healthcare utilisation, including hospitalisations and orthopaedic procedures, across different disease entities [4].

Nationwide administrative data provide a unique opportunity to assess temporal trends in disease burden and healthcare utilisation across large populations. In Germany, the Federal Health Monitoring system (GBE-Bund) offers comprehensive data on inpatient hospitalisations, enabling longitudinal analyses over extended time periods [5]. Complementary to this, the German Institute for the Hospital Remuneration System (InEK) provides detailed information on procedures performed during hospital stays through Diagnosis-Related Group (DRG) data. The combination of these data sources allows for a comprehensive evaluation of both epidemiological trends and surgical treatment patterns in routine clinical practice [6].

In addition to autoimmune inflammatory rheumatic diseases, osteomyelitis represents an important condition affecting bone health and requiring substantial healthcare resources. Although distinct in pathophysiology, osteomyelitis represents a musculoskeletal condition associated with substantial orthopaedic management and surgical burden. Including osteomyelitis as a comparator may provide additional insight into healthcare utilisation and orthopaedic care patterns across inflammatory and infectious musculoskeletal disorders [7, 8]. However, direct comparisons between osteomyelitis and inflammatory rheumatic diseases should be interpreted with caution because of differences in disease mechanisms, admission drivers, and treatment pathways.

Despite the availability of nationwide healthcare databases, comprehensive studies simultaneously evaluating long-term hospitalisation trends and orthopaedic care across multiple inflammatory rheumatic diseases remain scarce. Most existing investigations focus on individual diseases or specific outcomes, limiting direct comparisons across disease entities [9–11]. Understanding these patterns is essential for assessing the evolving burden of disease, identifying changes in healthcare delivery, and informing future planning in both rheumatology and orthopaedic surgery.

Therefore, the aim of this study was to analyse nationwide hospitalisation trends and associated orthopaedic surgical procedures in major inflammatory rheumatic diseases in Germany using nationwide hospital discharge data from GBE-Bund and DRG-based procedure data from InEK.

## Methods

### Study design and data source

This nationwide study was based on aggregated inpatient hospital discharge data from the German Federal Health Monitoring system (GBE-Bund) [5]. The GBE-Bund database provides comprehensive nationwide information on hospitalisations in Germany and allows for longitudinal analyses of healthcare utilisation over extended time periods.

To assess orthopaedic surgical procedures, data from the German Institute for the Hospital Remuneration System (InEK) were analysed using the Diagnosis-Related Group (DRG) browser. The InEK database contains procedure-specific information coded according to the German Procedure Classification (Operationen- und Prozedurenschlüssel, OPS) and reflects procedures performed during inpatient hospital stays [6].

### Study population

Hospitalisations associated with major inflammatory rheumatic diseases and osteomyelitis were identified using the International Classification of Diseases, 10th Revision, German Modification (ICD-10-GM). The following codes were included:

- Rheumatoid arthritis: M05–M06.
- Psoriatic arthritis: L40.5.
- Ankylosing spondylitis: M45.
- Juvenile arthritis: M08.
- Osteomyelitis: M86.

For analyses focusing on rheumatoid arthritis, codes M05.1x (rheumatoid lung disease), M05.2x (rheumatoid vasculitis), M06.1 (adult-onset Still disease), and M06.3 (rheumatoid nodules) were excluded to ensure a more homogeneous population representing classical rheumatoid arthritis. These exclusions were applied to reduce potential heterogeneity arising from extra-articular manifestations and related conditions. Hospitalisations were included if the respective ICD-10 code was recorded as the principal diagnosis. This approach was chosen to improve diagnostic specificity; however, disease-related admissions coded as secondary diagnoses were not captured.

### Data extraction

Annual numbers of inpatient hospitalisations for each disease group were extracted from the GBE-Bund database for the period 2010 to 2023. In addition, age-standardised hospitalisation rates per 100,000 population were obtained from the GBE-Bund database. These rates were provided by GBE-Bund and were standardised according to the German standard population 2022 [5]. Temporal trends were analysed descriptively across the study period.

For the assessment of surgical treatment patterns, orthopaedic procedures were identified using OPS codes within the InEK DRG browser. Procedure frequencies were extracted for cases in which the respective rheumatic disease was coded as the principal diagnosis. Procedures were analysed at the case level as reported by the InEK DRG browser. Separate queries were performed for each disease entity using the respective ICD-10-GM code as the principal diagnosis. Knee replacement procedures were identified using OPS code group 5–822, hip replacement procedures using OPS code group 5–820, and spine-related procedures in ankylosing spondylitis using selected OPS 5-83x codes. The corresponding OPS code groups used for procedure identification are provided in Supplementary Table S1. The DRG data correspond to DRG version 2024 and were grouped according to the 2025 classification. The DRG-based procedure analysis represents a cross-sectional assessment using the most recent available InEK dataset and was not intended to evaluate temporal changes in surgical procedures.

### Outcome measures

The primary outcome measures were:

1. Annual number of hospitalisations and age-standardised hospitalisation rates for each disease group.
2. Temporal trends in hospitalisation patterns between 2010 and 2023.
3. Disease-specific Frequency and distribution of orthopaedic procedure profiles.

Orthopaedic procedures of interest included joint replacement surgery (e.g., hip and knee arthroplasty) and spine-related surgical interventions (e.g., spinal stabilisation and spondylodesis).

### Statistical analysis

Descriptive statistical analyses were performed to evaluate temporal trends and differences between disease groups. Absolute case numbers were reported for each year and disease entity. Both absolute hospitalisation counts and age-standardised hospitalisation rates were analysed descriptively. Relative changes over time were calculated as percentage differences between baseline and final observation years. The analysis was descriptive in nature and intended to characterise national hospitalisation patterns rather than to perform inferential trend modelling. Consequently, no joinpoint analysis, annual percentage change calculations, or regression-based trend modelling were performed.

Due to the aggregated nature of the data, no individual-level statistical comparisons were performed. Data management and descriptive analyses were performed using standard spreadsheet-based software.

### Ethical considerations

The study was based exclusively on publicly available aggregated data and did not involve individual patient data. No individual-level or identifiable patient data were accessed or processed. The analysed data were obtained from publicly accessible national databases and consisted exclusively of aggregated statistics. Therefore, ethical approval and informed consent were not required in accordance with national regulations.

## Results

### Hospitalisation trends (2010–2023)

**Fig. 1:**
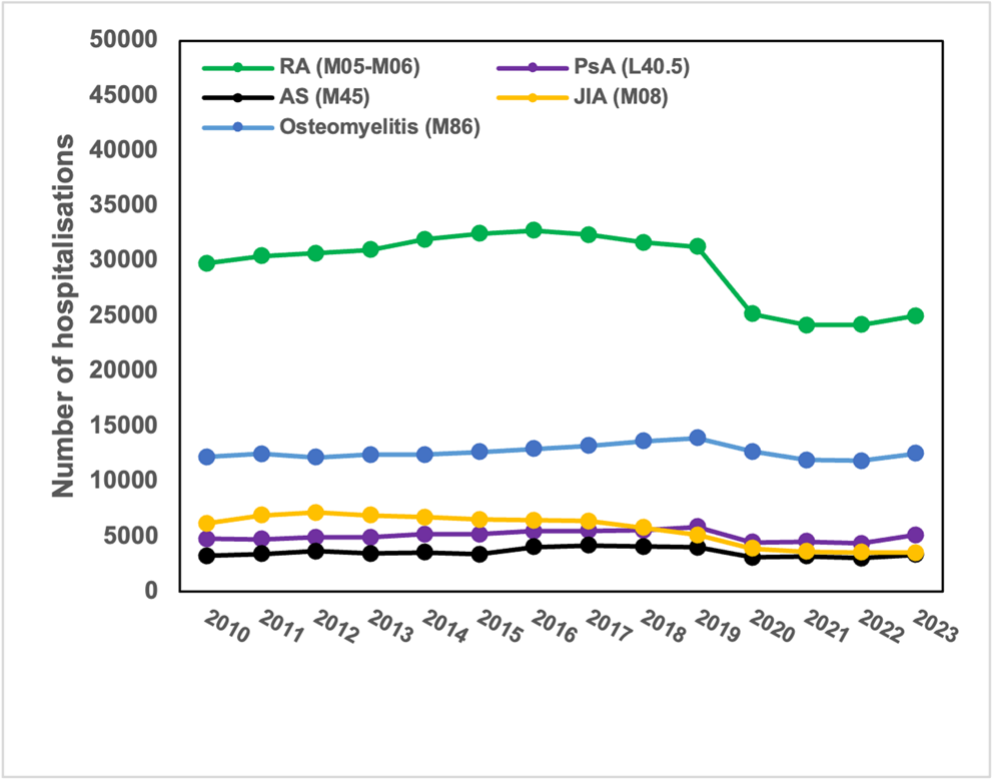
Temporal trends in hospitalisations for major inflammatory rheumatic diseases and osteomyelitis in Germany between 2010 and 2023. Annual inpatient hospitalisation numbers were obtained from the German Federal Health Monitoring system (GBE-Bund) using ICD-10-GM principal diagnoses for rheumatoid arthritis (RA; M05–M06), psoriatic arthritis (PsA; L40.5), ankylosing spondylitis (AS; M45), juvenile idiopathic arthritis (JIA; M08), and osteomyelitis (M86). Rheumatoid arthritis accounted for the highest number of hospitalisations throughout the study period. A marked decline in hospitalisations was observed across all disease groups in 2020, coinciding with the onset of the COVID-19 pandemic

Hospitalisation patterns differed substantially across disease groups during the study period (Fig. 1; Table 1). Rheumatoid arthritis (RA) accounted for the highest number of hospitalisations throughout the observation period. Hospitalisations increased from 29,775 cases in 2010 to a peak of 32,755 cases in 2016 before declining to 24,992 cases in 2023, corresponding to a 16.1% reduction compared with 2010.

**Table 1:** Hospitalisation trends in major inflammatory rheumatic diseases and osteomyelitis in Germany between 2010 and 2023

Disease	2010	2023	Relative change (%)
Rheumatoid arthritis (RA)	29,775	24,992	−16.1
Psoriatic arthritis (PsA)	4777	5138	+ 7.6
Ankylosing spondylitis (AS)	3252	3347	+ 2.9
Juvenile idiopathic arthritis (JIA)	6177	3521	−43.0
Osteomyelitis	12,234	12,538	+ 2.5

Annual numbers of inpatient hospitalisations were obtained from the German Federal Health Monitoring system (GBE-Bund) using ICD-10-GM codes for rheumatoid arthritis (M05–M06), psoriatic arthritis (L40.5), ankylosing spondylitis (M45), juvenile arthritis (M08), and osteomyelitis (M86). Relative changes were calculated as the percentage difference between 2010 and 2023. Negative values indicate a reduction in hospitalisations over time, whereas positive values indicate an increase

Juvenile arthritis (JIA) demonstrated the most pronounced decline, with hospitalisations decreasing from 6177 cases in 2010 to 3521 cases in 2023 (− 43.0%). In contrast, hospitalisations for psoriatic arthritis (PsA) increased from 4777 to 5138 cases (+ 7.6%), while ankylosing spondylitis (AS) remained largely stable, increasing slightly from 3252 to 3347 cases (+ 2.9%). Osteomyelitis also showed an overall stable pattern, increasing marginally from 12,234 to 12,538 cases (+ 2.5%), although a transient rise was observed between 2014 and 2019.

Across all disease groups, a marked reduction in hospitalisations was observed in 2020, coinciding with the onset of the COVID-19 pandemic and substantial disruptions in healthcare utilisation.

Age-standardised hospitalisation rates largely mirrored the trends observed for absolute hospitalisation counts (Table 2). Rheumatoid arthritis decreased from 40 to 30 hospitalisations per 100,000 population between 2010 and 2023, whereas juvenile idiopathic arthritis declined from 7 to 4 per 100,000 population. Psoriatic arthritis, ankylosing spondylitis, and osteomyelitis showed only minor fluctuations and remained largely stable over the study period.

**Table 2:** Age-standardised hospitalisation rates for major inflammatory rheumatic diseases and osteomyelitis in Germany between 2010 and 2023

Disease	ICD-10-GM	2010	Peak year	Peak rate	2023
Rheumatoid arthritis	M05 + M06	40	2016	41	30
Psoriatic arthritis	L40.5	6	2016–2019	7	6
Ankylosing spondylitis	M45	4	2016–2019	5	4
Juvenile idiopathic arthritis	M08	7	2012	9	4
Osteomyelitis	M86	16	2016–2019	17	15

Age-standardised hospitalisation rates are presented as cases per 100,000 population and were obtained from the German Federal Health Monitoring system (GBE-Bund). Rates were standardised according to the German standard population 2022. Peak year indicates the year with the highest observed age-standardised hospitalisation rate during the study period. Rheumatoid arthritis (RA) was defined by ICD-10-GM codes M05–M06, psoriatic arthritis (PsA) by L40.5, ankylosing spondylitis (AS) by M45, juvenile idiopathic arthritis (JIA) by M08, and osteomyelitis by M86

### Orthopaedic surgical procedures

**Fig. 2:**
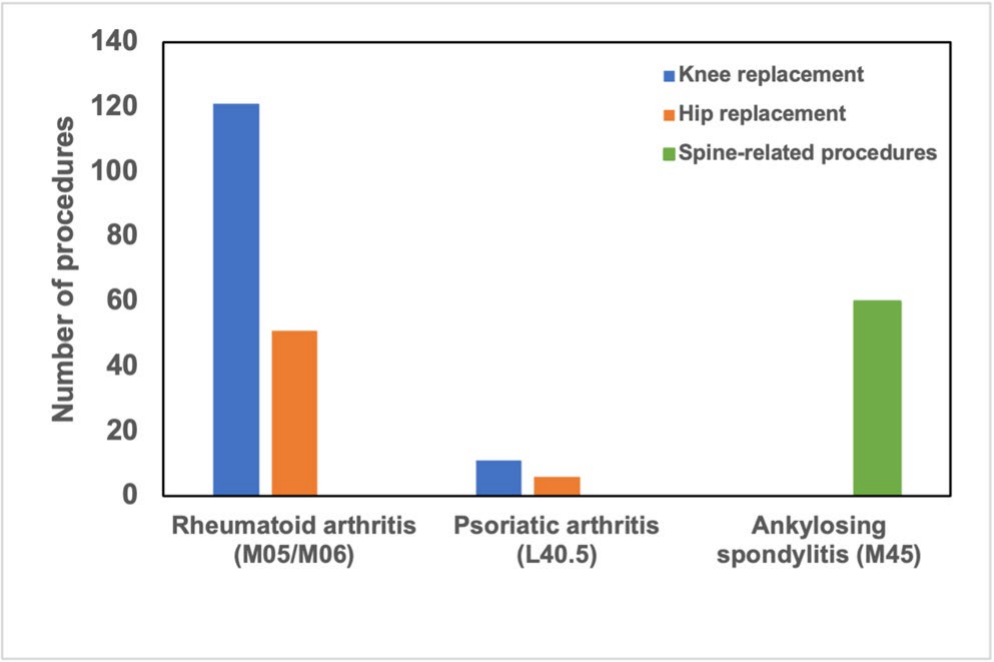
Major orthopaedic procedure profiles across inflammatory rheumatic diseases in Germany. Procedure frequencies were derived from the German Institute for the Hospital Remuneration System (InEK) DRG browser using disease-specific ICD-10-GM principal diagnoses and DRG version 2024 grouped according to the 2025 classification. Frequencies represent inpatient hospital cases. Rheumatoid arthritis (RA; M05–M06) was predominantly associated with knee replacement (*n* = 121) and hip replacement procedures (*n* = 51), whereas psoriatic arthritis (PsA; L40.5) showed substantially lower numbers of joint replacement procedures (11 knee and 6 hip replacements). Ankylosing spondylitis (AS; M45) was primarily associated with spine-related procedures (*n* = 60), reflecting the characteristic axial involvement of the disease

Analysis of cross-sectional DRG-based procedure data from the 2024 InEK dataset, grouped according to the 2025 classification, revealed distinct disease-specific orthopaedic procedure profiles (Fig. 2). Among hospitalised patients with RA (M05/M06), knee joint replacement (OPS 5–822; *n* = 121) and hip replacement (OPS 5–820; *n* = 51) represented the most frequently performed procedures.

In patients with PsA (L40.5), similar procedure profiles were observed but with substantially lower case numbers, including 11 knee replacements and 6 hip replacements. In contrast, AS (M45) was predominantly associated with spine-related surgical procedures, reflecting the characteristic axial involvement of the disease.

Overall, these findings demonstrate clear differences in surgical treatment patterns across inflammatory rheumatic diseases, corresponding to their distinct clinical manifestations.

## Discussion

### Interpretation of findings

The present nationwide analysis demonstrates heterogeneous developments in hospitalisation patterns across inflammatory rheumatic diseases. The observed decline in hospitalisations for rheumatoid arthritis is consistent with the paradigm shift in disease management over the past two decades. The introduction and widespread use of biologic and targeted synthetic disease-modifying antirheumatic drugs have significantly improved disease control, reduced inflammatory activity, and slowed structural joint damage. Although the present dataset does not permit causal inference, these therapeutic advances may have contributed to the decreasing need for inpatient care observed in patients with rheumatoid arthritis [12].

A similar trend was observed in juvenile arthritis, where the marked reduction in hospitalisations may also reflect earlier diagnosis, improved treatment strategies, and more effective outpatient management [13]. Importantly, age-standardised hospitalisation rates largely mirrored the trends observed for absolute hospitalisation counts, suggesting that demographic changes alone are unlikely to explain the observed developments.

In contrast, the relatively stable trends observed in ankylosing spondylitis and the moderate increase in psoriatic arthritis suggest that disease burden and healthcare utilisation remain substantial in these patient populations. These differences may be explained by variations in disease phenotype, treatment response, and the extent to which structural damage can be prevented by current therapies [14]. A pronounced decline in hospitalisations was observed across all disease groups in 2020. This finding likely reflects the impact of the COVID-19 pandemic on healthcare utilisation, including reduced hospital admissions, postponement of elective procedures, and changes in healthcare-seeking behaviour. Consequently, the observed reduction in 2020 should be interpreted within the context of pandemic-related healthcare disruptions rather than disease-specific effects alone.

### Comparison with previous literature

The findings of this study are in line with previous reports suggesting a decline in joint replacement rates among patients with rheumatoid arthritis in the era of modern pharmacological treatment [9]. However, prior studies have often been limited to specific regions or single-centre cohorts, whereas the present analysis provides a comprehensive nationwide perspective over an extended observation period [11].

Furthermore, Zhao et al. reported progressive large-joint involvement and surgical interventions in a longitudinal cohort of patients with rheumatoid arthritis [15]. While providing important disease-specific clinical insights, their study focused exclusively on rheumatoid arthritis, whereas the present analysis enables nationwide comparisons across multiple major inflammatory rheumatic diseases and integrates disease-specific orthopaedic procedure profiles derived from German DRG-based data.

Comparative data across different inflammatory rheumatic diseases remain scarce. To our knowledge, few studies have simultaneously compared nationwide hospitalisation patterns across multiple major inflammatory rheumatic diseases while also integrating disease-specific orthopaedic procedure profiles. By analysing multiple disease entities within a unified framework, this study contributes to a more differentiated understanding of disease-specific healthcare utilisation patterns. The observed stability in ankylosing spondylitis and the moderate increase in psoriatic arthritis hospitalisations are consistent with previous reports indicating persistent disease burden despite therapeutic advances [14].

### Clinical relevance

From a clinical perspective, the findings emphasise that, despite substantial progress in pharmacological treatment, orthopaedic surgery continues to play an essential role in the management of inflammatory rheumatic diseases [1]. The persistence of joint replacement procedures in rheumatoid arthritis and the need for spine surgery in ankylosing spondylitis highlight that structural damage remains clinically relevant, particularly in patients with long-standing disease or insufficient treatment response.

The analysis of orthopaedic surgical procedures further highlights disease-specific patterns. In rheumatoid arthritis, the predominance of joint replacement procedures, particularly of the knee and hip, reflects the typical peripheral joint involvement and long-term consequences of joint destruction [16]. In contrast, ankylosing spondylitis was primarily associated with spine-related surgical interventions, consistent with its characteristic axial involvement and the potential development of spinal deformities requiring surgical correction [17].

These results underline the importance of early and effective disease control to prevent irreversible structural damage and reduce the need for surgical interventions. At the same time, these findings emphasise the continued need for close collaboration between rheumatologists and orthopaedic surgeons in the management of complex cases.

### Implications for healthcare systems

The observed changes in hospitalisation patterns have important implications for healthcare systems. The marked decline in hospitalisations for rheumatoid arthritis and juvenile arthritis suggests a shift in disease management toward more effective outpatient care. Although causal relationships cannot be established from the present dataset, the widespread use of biologic and targeted synthetic therapies may have contributed to this development. This shift may reduce the burden on inpatient services but simultaneously increases the demand for specialized outpatient rheumatology care and long-term disease monitoring [18].

Osteomyelitis was included as a comparator musculoskeletal disorder rather than as an inflammatory rheumatic disease. Its inclusion was intended to provide contextual information on hospitalisation and orthopaedic care patterns in a condition associated with substantial musculoskeletal morbidity and surgical burden.

In contrast, the relatively stable or moderately increasing hospitalisation rates for psoriatic arthritis and ankylosing spondylitis indicate that inpatient care remains relevant for these patient populations. This may reflect differences in disease manifestations, treatment responses, or healthcare utilisation patterns, and highlights the need for disease-specific healthcare strategies [19].

From an orthopaedic perspective, the persistence of surgical interventions, particularly joint replacement in rheumatoid arthritis and spine-related procedures in ankylosing spondylitis, underscores the ongoing need for specialized rheuma-orthopaedic expertise. Despite advances in pharmacological therapy, structural damage requiring surgical treatment continues to occur, necessitating integrated care pathways between rheumatology and orthopaedic surgery [10].

Furthermore, an increasing proportion of orthopaedic procedures may be performed in outpatient settings, which is not captured by the present dataset. This may alter healthcare resource utilisation and should be considered when interpreting the present findings.

### Strengths and limitations

This study has several notable strengths. First, it is based on nationwide hospital discharge data, allowing for a comprehensive assessment of hospitalisation patterns across inflammatory rheumatic diseases in Germany over more than a decade. The use of the German Federal Health Monitoring system (GBE-Bund) ensures high coverage of inpatient cases and enables robust longitudinal analyses. Second, the combination of epidemiological data with DRG-based procedure data from the German Institute for the Hospital Remuneration System (InEK) provides a unique perspective by linking temporal trends in hospitalisations with disease-specific orthopaedic surgical patterns. This integrated approach allows for a more comprehensive evaluation of healthcare utilisation than studies relying on a single data source.

However, several limitations should be considered when interpreting the findings. The analysis is based on aggregated administrative data, which do not allow for patient-level analyses or adjustment for potential confounders such as disease severity, comorbidities, or treatment regimens. Furthermore, the analysed data represent hospitalisations rather than individual patients. Consequently, repeated admissions of the same patient may have contributed to the observed case numbers. In addition, only hospitalisations coded with the respective disease as the principal diagnosis were included. This approach improves diagnostic specificity but may underestimate disease-related hospitalisations and orthopaedic procedures performed during admissions primarily coded under other diagnoses. In addition, the identification of cases relies on ICD-10 coding, which may be subject to coding variability and misclassification. To address potential demographic influences, age-standardised hospitalisation rates provided by GBE-Bund were additionally analysed and showed patterns comparable to those observed for absolute hospitalisation counts.

Additionally, the DRG-based analysis primarily captures inpatient procedures and therefore does not capture orthopaedic interventions performed in outpatient settings. As a result, the overall surgical burden may be underestimated.

## Conclusions

Nationwide hospitalisation patterns for inflammatory rheumatic diseases in Germany have changed substantially over the past decade, with marked declines observed for rheumatoid arthritis and juvenile idiopathic arthritis. In contrast, psoriatic arthritis and ankylosing spondylitis showed more stable trends, indicating persistent inpatient care needs in these populations.

Despite these developments, orthopaedic surgery remains an important component of disease management. Distinct disease-specific surgical patterns, including joint replacement procedures in rheumatoid arthritis and spine-related interventions in ankylosing spondylitis, underline the different structural manifestations of inflammatory rheumatic diseases. The persistence of these patterns despite advances in medical therapy highlights the continuing importance of integrated rheumatologic and orthopaedic care.

Future studies should further investigate healthcare utilisation and surgical burden across both inpatient and outpatient settings to better inform clinical practice and healthcare planning.

## Supplementary Information

Below is the link to the electronic supplementary material.

Supplementary Material 1

## Data Availability

The data used in this study are publicly available, aggregated administrative data from the German Federal Health Monitoring System (GBE-Bund) [5] and the German Institute for the Hospital Remuneration System (InEK) [6]. No individual-level patient data were used.

## Declarations

### AI use statement

Artificial intelligence-assisted tools were used for language improvement and editorial support during manuscript preparation. All scientific content, study design, data collection, analyses, interpretation of results, and conclusions were developed, verified, and approved by the authors. The authors reviewed and edited the final manuscript and take full responsibility for its content.

### Disclaimer

This manuscript represents original work that has not been published previously and is not under consideration for publication elsewhere. All scientific content, analyses, interpretations, and conclusions were developed and verified by the authors, who take full responsibility for the integrity and accuracy of the work.

### Conference presentation

Part of this work has been accepted for presentation at the Deutscher Rheumatologiekongress 2026 (German Congress of Rheumatology), Leipzig, Germany, 9–12 September 2026, as Poster RO.06 in the session “PW25 – Rheuma-Orthopädie 1” under the title “Orthopaedic Surgical Patterns in Inflammatory Rheumatic Diseases in Germany: Nationwide Hospitalisation Trends and DRG-Based Procedure Analysis”. The present manuscript represents the full report of the study. At the time of submission of the revised manuscript, no bibliographic citation or DOI for the conference abstract was available.

### Supplementary Table S1. OPS code groups used for the orthopaedic procedure profile analysis

Procedure frequencies were extracted from the German Institute for the Hospital Remuneration System (InEK) DRG browser (DRG version 2024, grouped according to the 2025 classification) using disease-specific ICD-10-GM principal diagnoses. Rheumatoid arthritis (RA) was defined by ICD-10-GM codes M05 and M06, excluding M05.1x, M05.2x, M06.1, and M06.3. Knee replacement procedures were identified using OPS code group 5-822, hip replacement procedures using OPS code group 5-820, and spine-related procedures in ankylosing spondylitis (AS) using selected OPS 5-83x codes. Procedure counts represent case-level frequencies (“Fälle”) as reported by the InEK DRG browser. For RA, total procedure frequencies were calculated by combining the corresponding M05 and M06 results. Cases refer to inpatient hospital cases reported by the InEK DRG browser and not to individual patients.
