# Supplementary material for: Changing Patterns of Hospitalisation and Orthopaedic Procedure Profiles in Major Inflammatory Rheumatic Diseases in Germany: a Nationwide Analysis of Hospital Discharge Data
Source: Rheumatol Int. 2026 Jul 11;46(8):197. doi: 10.1007/s00296-026-06247-5 (PMC13356080; doi:10.1007/s00296-026-06247-5)
Supplement: Supplementary file 1 — Supplementary Material 1 [file 296_2026_6247_MOESM1_ESM.docx]

| **Disease** | **Procedure group** | **OPS code(s)** | **Cases** |
| --- | --- | --- | --- |
| RA (M05) | Knee replacement | 5-822.* | 96 |
| RA (M06) | Knee replacement | 5-822.g1, 5-822.j1 | 25 |
| RA (M05+M06) | Knee replacement | 5-822.* | 121 |
| RA (M05) | Hip replacement | 5-820.* | 31 |
| RA (M06) | Hip replacement | 5-820.00, 5-820.02 | 20 |
| RA (M05+M06) | Hip replacement | 5-820.* | 51 |
| PsA (L40.5) | Knee replacement | 5-822.g1 | 11 |
| PsA (L40.5) | Hip replacement | 5-820.00 | 6 |
| AS (M45) | Spine-related procedures | 5-835.9, 5-83b.57, 5-832.1, 5-83w.0, 5-832.4, 5-832.5, 5-836.38, 5-83b.56 | 60 |

**Supplementary Table S1. OPS code groups used for the orthopaedic procedure profile analysis.** Procedure frequencies were extracted from the German Institute for the Hospital Remuneration System (InEK) DRG browser (DRG version 2024, grouped according to the 2025 classification) using disease-specific ICD-10-GM principal diagnoses. Rheumatoid arthritis (RA) was defined by ICD-10-GM codes M05 and M06, excluding M05.1x, M05.2x, M06.1, and M06.3. Knee replacement procedures were identified using OPS code group 5-822, hip replacement procedures using OPS code group 5-820, and spine-related procedures in ankylosing spondylitis (AS) using selected OPS 5-83x codes. Procedure counts represent case-level frequencies (“Fälle”) as reported by the InEK DRG browser. For RA, total procedure frequencies were calculated by combining the corresponding M05 and M06 results. Cases refer to inpatient hospital cases reported by the InEK DRG browser and not to individual patients.
